# Non-Pharmacological Intervention for Personalizing Sleep Quality through Gentle Rocking Motion

**DOI:** 10.3390/jpm14020218

**Published:** 2024-02-19

**Authors:** Damiana-Maria Vulturar, Liviu-Ștefan Moacă, Ioana Maria Chețan, Ștefan Cristian Vesa, Teodora-Gabriela Alexescu, Cristina Grigorescu, Antigona Carmen Trofor, Mirela-Anca Stoia, Alexandra Floriana Nemes, Doina-Adina Todea

**Affiliations:** 1 Department of Pneumology, Iuliu Hatieganu University of Medicine and Pharmacy, 400332 Cluj-Napoca, Romania; vulturar.damianamaria@elearn.umfcluj.ro (D.-M.V.); mariaioana_25@yahoo.com (I.M.C.); dtodea@umfcluj.ro (D.-A.T.); 2 Pharmacology, Toxicology and Clinical Pharmacology Department, Iuliu Hațieganu University of Medicine and Pharmacy, 400337 Cluj-Napoca, Romania; stefanvesa@gmail.com; 3 4th Department Internal Medicine, “Iuliu Hatieganu” University of Medicine and Pharmacy, 400015 Cluj-Napoca, Romania; teodora.alexescu@umfcluj.ro (T.-G.A.); mirelastoia@yahoo.com (M.-A.S.); 4 Discipline of Pneumology, III-rd Medical Department, Faculty of Medicine, “Grigore T. Popa” University of Medicine and Pharmacy, 700115 Iasi, Romania; cristina.grigorescu@umfiasi.ro (C.G.); antigona.trofor@umfiasi.ro (A.C.T.); 5 Department of Cardiology, Emergency County Clinical Hospital, 400006 Cluj-Napoca, Romania; 6 Doctoral School, Faculty of Medicine, “Titu Maiorescu” University, 031593 Bucharest, Romania; alexandra.nemes@prof.utm.ro

**Keywords:** rocking motions, sleep architecture, Inoveris device, personalized sleep, non-pharmacological intervention

## Abstract

Introduction: Achieving restorative sleep is crucial for overall well-being, yet sleep difficulties affect a substantial portion of the adult population. Sleep disturbances are associated with diminished quality of life, physical complaints, cognitive impairment, and emotional regulation challenges. Objective: This study explores the influence of an innovative experimental bed designed to generate rocking motions on sleep parameters. Methods: A prospective observational study enrolled 60 adult participants, assessing their sleep on a regular stationary bed and the Inoveris bed, providing gentle rocking movements. Polysomnography was conducted, recording electroencephalography, electrooculogram, electromyogram, respiratory effort, and other parameters. Results: The rocking bed significantly increased total sleep time (TST) and reduced N1 sleep stage duration (*p* < 0.001). Participants also experienced a quicker transition to the N2 sleep stage (*p* = 0.01), indicative of a faster shift from wakefulness to deeper sleep. Additionally, rocking led to a higher percentage of N1 sleep stages (*p* = 0.01) and a significant increase in N3 sleep stage duration (*p* = 0.004). While some results lacked statistical significance, notable trends in the rocking bed group have clinical relevance, consistently improving sleep parameters, including increased TST. The rocking bed also showed a trend towards higher sleep efficiency (SE) and sleep duration percentage, hinting at a potential overall enhancement in sleep quality. Conclusion: This study contributes valuable insights into the potential benefits of rocking motions on sleep architecture. Despite variations in outcomes across studies, our results underscore the potential of rocking beds as a non-pharmacological intervention for enhancing sleep quality. Notable improvements in total sleep time (TST), N1 sleep stage reduction, and accelerated transitions to deeper sleep stages highlight the clinical relevance of rocking interventions. Further research, collaboration, and addressing the identified limitations will advance our understanding of the therapeutic applications of rocking motions in sleep science.

## 1. Introduction

While achieving restorative sleep is essential for overall well-being and daytime functionality, it often presents a challenge. Occasional sleep difficulties are widespread, affecting approximately one-third of the adult population [1]. Impaired sleep is linked to diminished quality of life and physical complaints, such as fatigue, headaches, dry and irritated eyes, or weight gain. It also impairs cognitive function and has a negative impact on emotion regulation. Poor sleep quality has been indicated to correlate with systemic inflammation and subclinical arterial diseases, and has been linked to the occurrence of heart failure in general populations, an unfavourable prognosis in patients with coronary disease, and overall mortality in a male general population [2,3,4,5].

Data from the Centers for Disease Control and Prevention revealed a consistent decrease in mean sleep duration and an increase in the percentage of adults sleeping ≤6 h per day between 1985 and 2012. This trend underscores the influence of social, lifestyle, and environmental factors on sleep patterns [6]. Given the rising prevalence of sleep-related issues and their implications for health and well-being, effective treatment of sleep disturbances is highly pertinent [7,8,9].

Pharmacological treatments pose a significant risk of dependency and frequently result in sedation the subsequent day, elevating the likelihood of falls and hip fractures, especially in the elderly population [10,11]. Considering these effects, non-pharmacological interventions are gaining interest. Relaxation techniques, warm feet, and music are popular methods in the general population to improve sleep quality [12]. The outcomes of a review and meta-analysis, which evaluated interventions including exercise, aromatherapy, acupressure, cognitive behavioural therapy, and meditation, indicated statistically significant effects on sleep [13].

Additionally, what prompts us to gently hold infants or succumb effortlessly to sleep in a hammock? While these uncomplicated actions are prevalent among various cultures and have persisted through generations, the underlying connection between the act of rocking and its influence on sleep remains inadequately comprehended.

Gentle rocking motions as a form of vestibular stimulation have emerged as a promising non-pharmacological option [14,15]. The utilization of vestibular stimulation has been explored as a soothing and calming intervention in the context of treating diverse psychiatric and neurological conditions [16]. Various studies have approached the connection between vestibular stimulation and sleep. It has been suggested that passive motions could be used to modify and potentially enhance sleep patterns.

Kompotis et al., in his study regarding an animal model, explored the impact of rocking on sleep by focusing on the vestibular system’s role. In this animal study, mice underwent continuous rocking for 12 h through a horizontally moving platform. The findings indicated that at the ‘optimal’ rate of 1 Hz, mice exhibited increased an NREM sleep duration compared to wakefulness, and rocking also hastened the onset of sleep [17].

The in-depth investigation into the impact of rocking on infant sleep originated in the 1970s and 1980s, utilizing oscillating waterbeds. Early studies yielded promising results, indicating that rocking expedited the initiation of sleep and fostered more extended, uninterrupted periods of rest [18].

The most complex diagnostic tool in sleep medicine is represented by polysomnography, a comprehensive sleep study that registers electroencephalography, electrooculogram, electromyogram, limb movements, heart rhythm, thoracic and abdominal respiratory effort, oxygen saturation, breath rate, and breathing patterns and monitors nasal or mouth airflow. In specially designed environments, the night can also be video-registered for a better comprehension of the events. This combination of techniques is utilized to investigate the underlying reasons behind disruptions in sleep. PSG is widely acknowledged as the golden standard for diagnosing sleep-related breathing disorders, encompassing conditions such as obstructive sleep apnoea (OSA), central sleep apnoea, and sleep-related hypoventilation or hypoxia. Furthermore, PSG can also serve as a tool to assess various other sleep disorders, including nocturnal seizures, narcolepsy, periodic limb movement disorder, and rapid eye movement sleep behaviour disorder [19]. Sleep apnoea is a sleep disorder characterized by interruptions in breathing during sleep, leading to brief and repetitive periods of oxygen deprivation. These pauses in breathing can last for seconds to minutes and may occur multiple times throughout the night [20]. While sleep apnoea is primarily recognized as a sleep disorder characterized by breathing interruptions, emerging research suggests that it also has significant metabolic implications, classifying it as a metabolic disease. Sleep apnoea can disrupt the balance of hormones that regulate metabolism, such as insulin and cortisol. The repeated drops in oxygen levels and fragmented sleep patterns associated with sleep apnoea can contribute to insulin resistance, potentially leading to type 2 diabetes. Additionally, sleep apnoea has been linked to alterations in appetite-regulating hormones, often resulting in increased cravings for high-calorie foods. Furthermore, the chronic inflammation associated with untreated sleep apnoea may contribute to metabolic dysfunction and cardiovascular complications. Recognizing sleep apnoea as a metabolic disease underscores the importance of holistic approaches to its management, including lifestyle modifications, weight management, and addressing underlying metabolic factors. Treating sleep apnoea not only improves sleep quality but also plays a crucial role in mitigating its impact on metabolic health [21].

Studies published investigating the possible influence of rocking movements on sleep architecture (rapid eye movement (REM) and non-rapid eye movement (nREM)), respiratory function (apnoea–hypopnea index (AHI) and saturation parameters), and movement disorders are rare and have certain limits: their small sample sizes, heterogenicity in study design, variability in rocking frequency, and lack of standardized definitions of parameters used.

Our primary objective is to study the influence of an innovative experimental bed designed to generate rocking motions on sleep parameters.

## 2. Materials and Methods

### 2.1. Study Design

#### 2.1.1. Participants

This study adopted a prospective observational design to investigate the impact of an innovative experimental bed generating rocking motions on sleep parameters. The choice of a prospective observational approach was grounded in its ability to capture real-time data, enabling a thorough examination of participant experiences in a naturalistic setting. This design choice allowed us to observe sleep patterns and potential improvements objectively, without intervention, offering valuable insights into the practical implications of rocking motions on sleep quality.

The inclusion and exclusion criteria for participant selection were meticulously defined to ensure the study’s integrity and the reliability of the obtained results. A cohort of 60 adult volunteers participated in the study, recruited from the Department of Somnology at the University of Medicine and Pharmacy, “Iuliu Hatieganu” Cluj-Napoca, Romania, between July 2022 and April 2023. The inclusion criteria required participants to be free from chronic conditions, aged 18 years or older, and willing to provide written informed consent for a polysomnographic evaluation.

Participants were excluded from the study if they presented neurological disorders and/or neuropathic pain, experienced active inflammation or confirmed active malignancy, suffered from severe respiratory or cardiac conditions, underwent treatments affecting muscle function and sleep architecture, had severe mental disorders, displayed cognitive disabilities, or demonstrated non-compliance during the study duration.

#### 2.1.2. Polysomnography

All the subjects included (n = 60) underwent polysomnographic investigations with a Philips Respironics Alice 6 LDx polysomnography device.

Each subject underwent two recordings, one in a normal bed and one recording with the Inoveris dispositive (a rocking bed). The recordings were conducted between 22:00 and 6:00, in accordance with the patient’s natural circadian rhythm. Sleep time was spent in complete darkness in a controlled room temperature (21 ± 1 °C) and the level of auditory stimulation was around 37 dB. All the electrodes (EEG, EOG, EMG, and ECG) were placed in line with guidelines set out by the American Academy of Sleep Medicine (AASM) [22] together with thoracic and abdominal belts recording breathing activity. Sleep parameters were obtained and classified according to standard AASM criteria [22].

During both sessions, polysomnography data were recorded continuously and analysed by medical professionals (experienced polysomnography scorers).

#### 2.1.3. Sleep Parameters

Different sleep parameters, such as TIB (time in bed), representing the total duration spent in bed during experiments; TST (total sleep time); and TSP (total sleep period), were computed for the quantitative assessment of sleep quality. Time in bed was calculated by considering lights-off time and lights-on time. TST is the summation of the entire duration spent asleep across all sleep stages, excluding wakefulness. On the other hand, TSP denotes the actual time spent in sleep cycles, calculated from the duration between the onset of the initial N1 sleep stage and waking up. Sleep efficiency is the proportion of sleep that takes place during the total sleep period (%TSP). Further exploration into sleep parameters included a focus on deep sleep periods such as N3, as well as lighter sleep and wake periods represented by the N1, N2, and REM stages. To ensure a more precise analysis, this study also considered parameters like the length of the N1, N2, N3, and REM sleep stages and the ratios of N1, N2, N3, and REM to TST (N1%, N2%, N3%, and REM%). The time from turning off the lights until the occurrence of two consecutive epochs of the relevant sleep stage defined the latency to stages N1, N2, and N3.

#### 2.1.4. Inoveris Device

**Figure 1:**
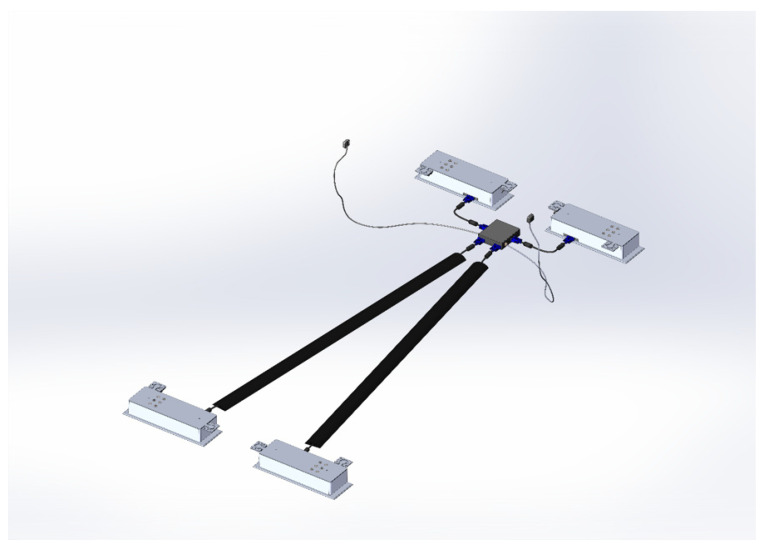
Schematic representation of the Inoveris device.

**Figure 2:**
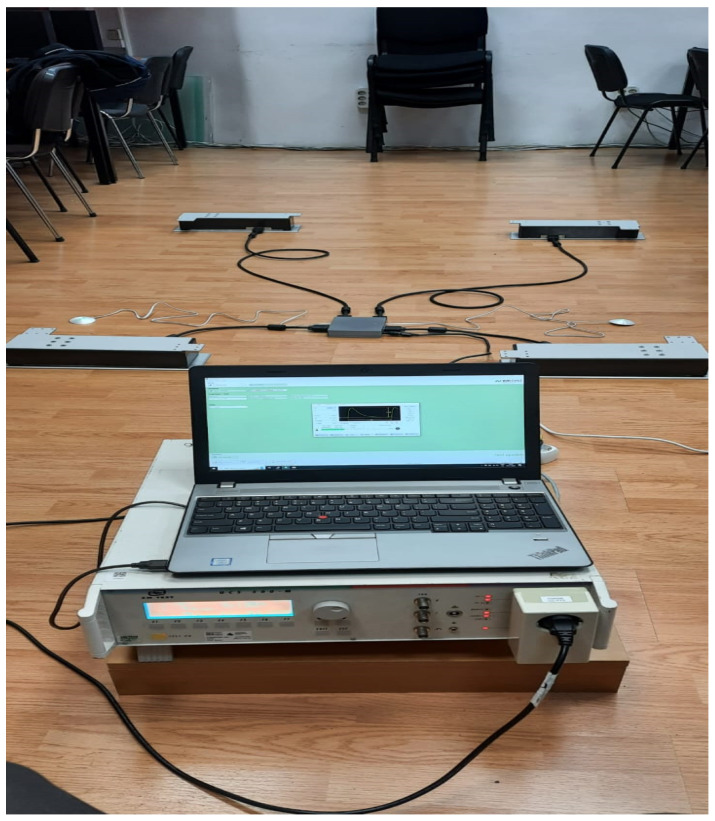
The technology of the Inoveris device.

**Figure 3:**
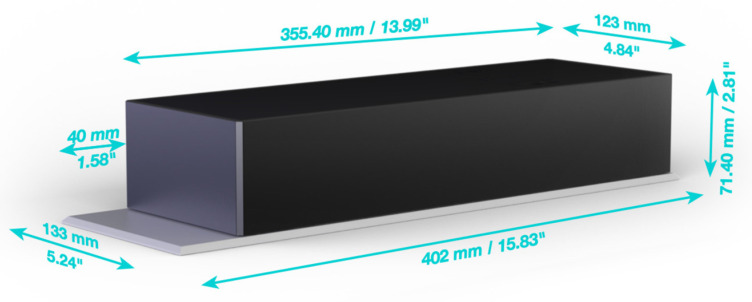
Mechanical design of a leg in the Inoveris device.

The Inoveris device is as intricate as the drivetrain of a four-wheel-drive electric car, employing high-quality silent servomotors—one for each leg. The apparatus comprises four modular supporting legs (Figure 1), a control unit that synchronizes leg motions, a remote control, two wired motion sensors to be affixed to the bed mattress, and a smartphone app for advanced control. The Inoveris device applies a slow, oscillating motion to the bed, facilitated by an integrated and programmable motion control system. The technology utilizes custom-manufactured electro-mechanical components to generate a gentle and nearly silent motion. Electrical specifications include a power consumption range (load-dependent) from 10 W to 20 W; a standby power consumption of <3 W; WIFI and Bluetooth connectivity; and an AC/DC power supply unit of 12 V, 80 W (Figure 2). Mechanical features encompass robust legs capable of supporting a bed, including occupants weighing up to 1000 kg/2200 lbs, a motion oscillation range of 4 cm/1.6”, and whisper-quiet 20 dBA actuators. The leg dimensions are 42 cm (L) × 13.3 cm (W) × 71.4 cm (H)/15.83 in (L) × 5.24 in (W) × 2.81 in (H), with a leg weight of 7.5 kg/16.5 lbs and a total leg weight for all four units of 30 kg/66 lbs (as shown in Figure 3).

**Figure 4:**
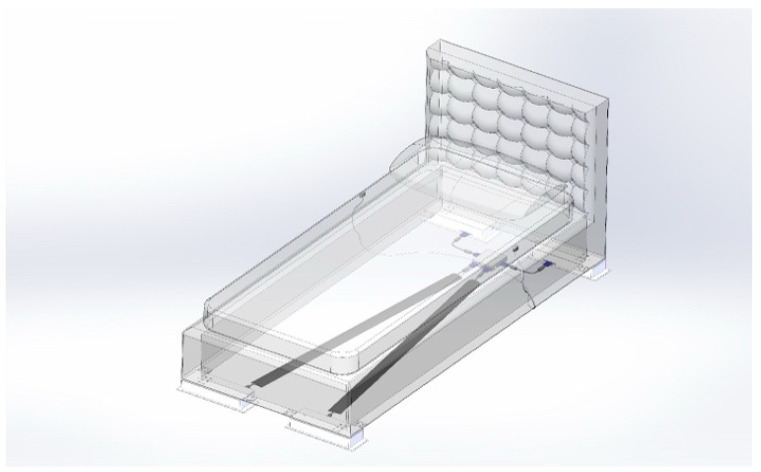
Schematic representative of rocking motion with the Inoveris device.

**Figure 5:**
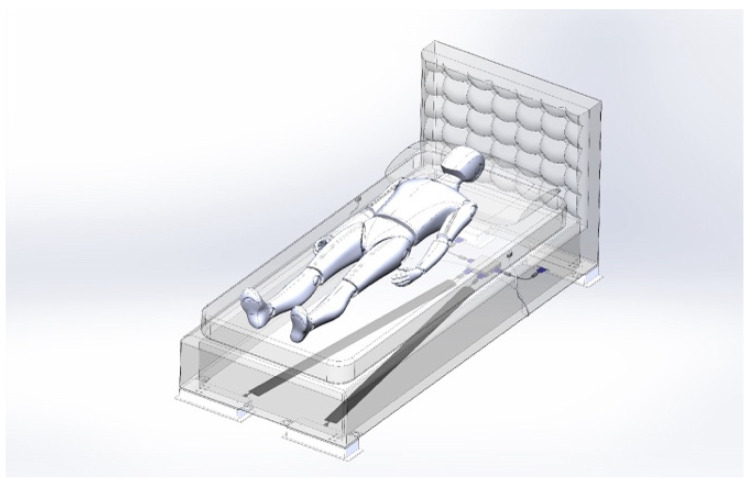
Schematic representative of a rocking motion with the Inoveris device.

Rocking motion features (Figure 4 and Figure 5).

The rocking motion path length of the Inoveris device is 40 mm, and all movements are executed with an amplitude of 40 mm. It is recognized that an S-shaped motion profile yields a considerably smoother return at the end of the path, making it imperceptible to the user compared to a linear or trapezoidal motion curve. The S-shaped motion profile involves variable (non-linear) acceleration and deceleration. The slew speed represents the maximum travel speed. The rocking motion oscillation frequency is adjustable between 0.05 and 0.25 Hz, influencing the slew speed and the corresponding length of the S-shaped acceleration and deceleration segments. Experimental observations indicate that a longer S-shaped acceleration interval, indicating a higher speed variation throughout the entire motion path, enhances the effectiveness of the rocking motion outcome.

### 2.2. Statistical Analysis

Statistical analysis was performed using MedCalc^®^ Statistical Software version 22.016 (MedCalc Software Ltd., Ostend, Belgium; https://www.medcalc.org; 2023). To accurately represent the non-normally distributed data (tested with the Shapiro–Wilk test), the quantitative variables were summarized as median and interquartile range (25–75 percentiles), while the qualitative variables were described using frequency and percentage distributions. The choice of these summary statistics was guided by the nature of the data and aimed to provide a comprehensive depiction of the dataset. Comparisons between the measurements were carried out utilizing the Wilcoxon test. This non-parametric test was selected due to the non-normal distribution of our quantitative data and its robustness against outliers. By employing the Wilcoxon test, we aimed to ensure the validity of our statistical comparisons despite potential deviations from normality. A significance level of *p* < 0.05 was adopted to determine statistical significance. This threshold was chosen to balance between the risk of Type I and Type II errors and aligns with standard practice in hypothesis testing. While recognizing the importance of controlling for Type I errors, we deemed a *p*-value threshold of 0.05 appropriate for our exploratory analysis, allowing for the detection of meaningful differences in our dataset.

## 3. Results

The participants (n = 60), median age 26 years with no history of sleep disorders, spent one night in the normal bed and one night in the Inoveris dispositive. The results are presented in Table 1. The parameters are categorized into four groups: sleep stage (min), representing the duration of each sleep stage; sleep stage (%), indicating the percentage of each sleep stage relative to the total sleep length; sleep latencies, denoting the time taken to enter specific sleep stages; and sleep indexes, encompassing sleep parameters recommended by the American Association of Sleep Medicine (AASM).

The rocking bed demonstrated a statistically significant increase in total sleep time (TST), with a notably reduced duration of N1 sleep stages (*p* < 0.001). Additionally, subjects experienced a significant decrease in the latency for entering an N2 sleep stage (*p* = 0.01), highlighting a quicker transition from wakefulness to a deeper sleep state. Rocking also resulted in a higher percentage of N1 sleep stages (*p* = 0.01) and a significant increase in N3 sleep stage duration (*p* = 0.004). While some results did not reach statistical significance, it is important to note notable trends in the rocking bed group that could still be of clinical relevance. The rocking bed demonstrated a consistent improvement in sleep parameters, showcasing an increased total sleep time (TST) compared to the normal bed. Furthermore, the rocking bed exhibited a trend towards higher sleep efficiency (SE) and sleep duration percentage, reflecting a potential enhancement in overall sleep quality.

## 4. Discussion

Our investigation into the influence of rocking movements on sleep architecture yielded significant insights, aligning with and extending upon findings from prior studies. Perault et al.’s study involving 18 healthy participants revealed a notable increase in the duration of the N3 sleep stage during nights with rocking, consistent with our observations. This augmentation in deep sleep stages suggests a potential enhancement in sleep quality and restorative functions [23].

In parallel, Baek et al.’s exploration of rocking movements during three-hour naps in 15 young patients showed similarities to our findings, particularly regarding the increased duration of deep sleep (N3) and a significant reduction in the N1 stage (*p* < 0.005). The decreased latency period to enter deep sleep and the heightened frequency of sleep spindles align with our results, supporting the notion that rocking motions positively impact the sleep transition process [24].

The study by Bayer et al. including 12 male patients, focusing on the effects of reclining on a gently oscillating bed, parallels our findings of a facilitated transition from wakefulness to sleep, prolonged duration of N2 sleep stages, and an increased spindle density (*p* < 0.001). These similarities further strengthen the evidence suggesting a favourable impact of rocking beds on sleep architecture [14].

However, a nuanced perspective emerges when considering the study by Sluijs et al. in a study of 22 young patients, which investigated the effects of rocking beds with varying intensity on naps (45 min). While our results echoed a trend towards increased consolidated sleep during movement naps, this effect did not reach statistical significance. The absence of discernible differences in slow waves and sleep spindles during stage N3 suggests a complex relationship between rocking intensity and sleep architecture, warranting further exploration [25].

However, Sluijs et al., who included 19 patients with no sleep disorders and a mean age of 66.7 years, assessed the influence of an entire night of sleep in a rocking bed and provided contrasting findings. Unlike our study, they did not observe enhancements in sleep onset, increased deep sleep, or a rise in the quantity or density of sleep spindles. The diminished depth of sleep and the tendency towards a decrease in spindle amplitude, without impacting memory performance, highlighted the variability in outcomes associated with rocking bed stimulation [26].

The clinical relevance of our study lies in its contribution to sleep science and non-pharmacological interventions. Improved sleep parameters and enhanced sleep architecture with rocking motions suggest the potential utility of this non-invasive approach for addressing sleep disturbances. Our findings, particularly the positive effects on sleep architecture, pave the way for further exploration into personalized sleep interventions. Deeper sleep stages, such as N3, are known for their restorative functions, aligning with the observed improvements in our study. As sleep quality is pivotal for overall well-being, our insights may inform future therapeutic strategies, emphasizing the potential of rocking interventions in improving sleep outcomes.

Practical applications of these findings in clinical settings involve understanding the neural connections established by vestibular and sensory receptors with cortical structures associated with emotions. These connections play a role in various sleep aspects, and the inputs from vestibular or somatosensory systems facilitated by rocking movements may influence sleep control centres. Our study contributes to the unravelling of these mechanisms, enhancing our understanding of the clinical applications of rocking motions in managing sleep-related challenges [27,28,29,30]. Notably, Omlin et al.’s study on memory performance improvement over a night of sleep with lateral rocking movements provides additional support for the clinical potential of rocking interventions, particularly in individuals with poor baseline sleep [15].

These collective findings underscore the multifaceted nature of the impact of rocking motions on sleep architecture. While our results align with some studies, variations in outcomes across different investigations emphasize the need for more comprehensive research to elucidate the factors contributing to the effectiveness of rocking beds in promoting restful and restorative sleep. Future studies should consider variations in rocking intensity, duration, and individual differences to provide a more nuanced understanding of this promising area in sleep science.

## 5. Limitations

Despite the valuable insights gained from our study, several limitations should be acknowledged. The first limitation is represented by the small sample size. This limitation could impact the generalizability of our findings to broader populations. The small sample size might not fully capture the diversity present in larger, more heterogeneous populations. Consequently, caution should be exercised when extrapolating our results to different age groups, health conditions, or cultural backgrounds.

Secondly, the inclusion of participants from diverse age groups and health statuses, as observed in the referenced studies, introduces variability that may affect the consistency of observed effects. The potential differences in sleep patterns and responses to rocking interventions among participants with varying health conditions or age ranges could influence the generalizability of our results. Addressing this limitation could involve conducting more targeted studies focusing on specific age groups or health conditions to provide a clearer understanding of how rocking motions impact different populations. The subjective nature of sleep experiences and the potential influence of individual preferences on rocking bed efficacy further warrant consideration. Sleep experiences can vary significantly among individuals, and personal preferences may affect the perception of the rocking intervention. This subjectivity introduces variability that could impact the reproducibility of results across different individuals. Future studies may benefit from incorporating objective measures and exploring the role of individual preferences in more detail to enhance the reliability of the findings. Furthermore, the lack of standardized definitions and protocols in the existing literature makes direct comparisons challenging. Future studies should address these limitations by employing larger and more homogeneous samples, incorporating objective measures, and establishing standardized methodologies.

While our investigation yielded valuable insights, several challenges should be acknowledged. Participant adherence to the rocking protocol varied, impacting the consistency of rocking intensity across sessions. Standardizing rocking movements posed a challenge, as individual preferences and expectations influenced the experience. Technical issues related to the experimental bed’s functionality also arose, requiring adjustments during the study. Furthermore, managing participant expectations regarding the potential benefits of rocking motions introduced an additional layer of complexity. These challenges, while addressed to the best extent possible, may have influenced the outcomes and should be considered in interpreting the study’s findings.

## 6. Conclusions

Our study provides valuable contributions to the understanding of the impact of rocking motions on sleep architecture, revealing consistent improvements in sleep parameters with the use of a rocking bed. While our results align with some of the existing literature, variations across studies underscore the need for further research to elucidate the nuanced relationship between rocking movements and sleep quality. The promising outcomes observed in our investigation encourage continued exploration of rocking beds as a non-pharmacological intervention for promoting restful and restorative sleep. As we navigate this evolving field, addressing the identified limitations and fostering collaboration in research efforts will contribute to a more comprehensive and robust understanding of the therapeutic potential of rocking motions in sleep science.

## Figures and Tables

**Table 1:** Comparisons of the sleep parameters between control sleep and rocking bed.

Characteristics	Normal Bed	Rocking Bed	*p*
Time in bed (minutes)	480	480	
Total sleep period (TSP)	437 (378; 477)	430 (350; 443)	0.15
Total sleep time (TST)	336 (216; 404)	347 (279; 422)	0.036
Sleep efficiency (SE)	68.5 (57; 77)	80 (56; 84)	0.3
Sleep duration (%)	83.5 (71; 93)	90 (63; 94)	0.6
REM duration (%)	12.5 (9; 17)	14.5 (10; 18)	0.3
NREM duration (%)	71.5 (63; 75)	74 (60; 78)	0.3
**Sleep length (minutes)**			
N1 duration	49.5 (32; 64)	26.5 (16; 51)	<0.001
N2 duration	198 (154; 240)	168 (114; 232)	0.002
N3 duration	33 (15; 56)	42 (24.76)	0.004
REM duration	54 (32; 72)	60.5 (26; 67)	0.3
**Sleep latencies (minutes)**			
To N1	47 (29; 108)	9.5 (7; 16)	0.3
To N2	70.5 (42; 115)	58 (32; 69)	0.01
To N3	90 (46; 154)	80.5 (59; 108)	0.6
**Sleep Ratio (%) of TST**			
N1%	15.5 (11; 22)	48.5 (28; 66)	0.01
N2%	56.5 (52; 65)	54 (48; 61)	0.01
N3%	10.5 (5; 19)	16.5 (8; 22)	0.01
REM %	15.5 (12; 19)	16.5 (14; 21)	0.2

Data are presented as n (%) or median and 25–75 percentiles.

## Data Availability

The data are not publicly available due to data protection regulations.
